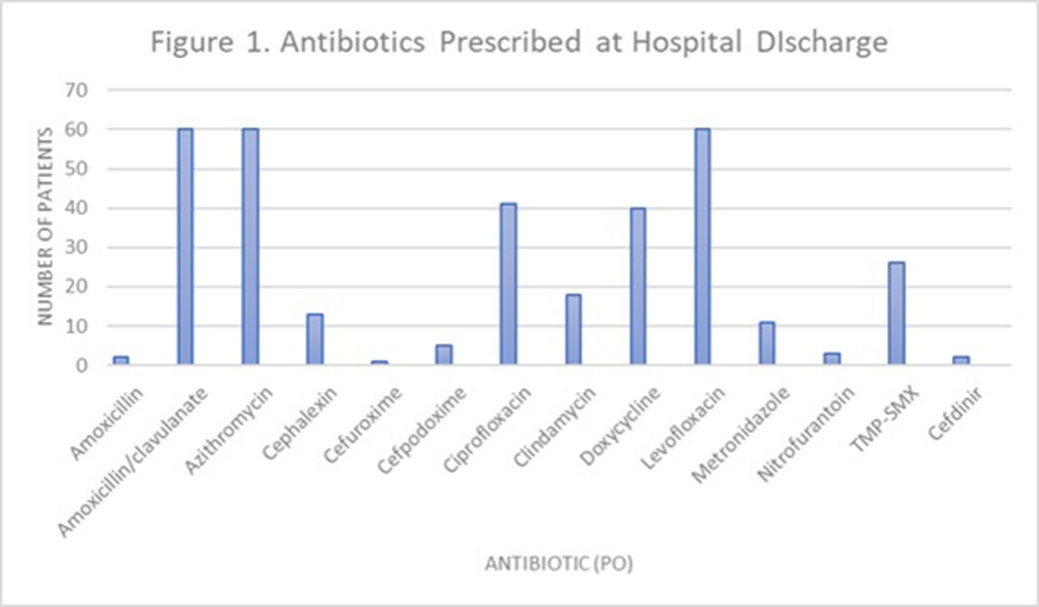# Appropriateness of Antibiotic Duration at the Time of Hospital Discharge

**DOI:** 10.1017/ash.2021.54

**Published:** 2021-07-29

**Authors:** Carly Sedlock, Jason Gallagher, Marissa Cavaretta, Alexander Haines, Kevin Nguyen, Neelesh Agarwal

## Abstract

**Background:** Antimicrobial stewardship initiatives usually occur in the inpatient setting, and they are often lacking at transitions of care (TOC), including hospital discharge. We assessed the appropriateness of antibiotic treatment duration at the time of discharge from our institution. **Methods:** This retrospective chart review included 300 adult patients discharged on oral antibiotics for acute infections during a 3-month period in 2019. The primary outcome was the duration of antibiotic therapy (DOT). To assess appropriateness, we compared the prescribed DOT (1) to that recommended by clinical guidelines, (2) to the minimum supported by clinical trials, and (3) to the period beyond the point of clinical stability, defined as normal vital signs with improvement in symptoms present from diagnosis. Each indication and antibiotic was assessed using standards appropriate for the combination. **Results:** Results are shown in Tables 1 and 2 and Figure 1. **Conclusions:** Antibiotics were often given longer than necessary on hospital discharge. In this study, patients received a median 2 days of excess antibiotics compared to recommended guidelines and 6 days after reaching clinical stability. A pilot TOC stewardship program was initiated to address this problem.

**Funding:** No

**Disclosures:** None

**Table 1.**
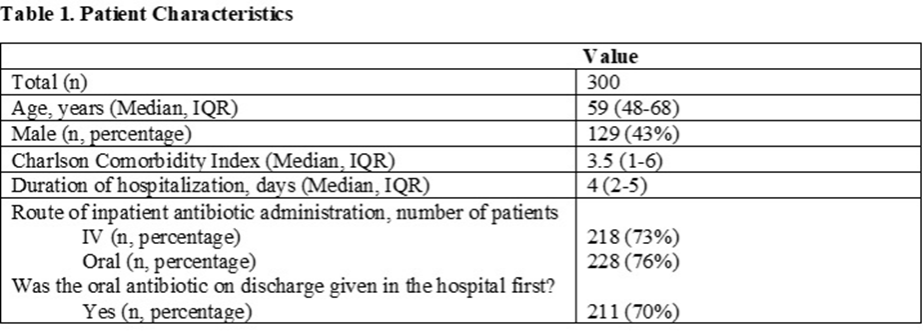

**Table 2.**
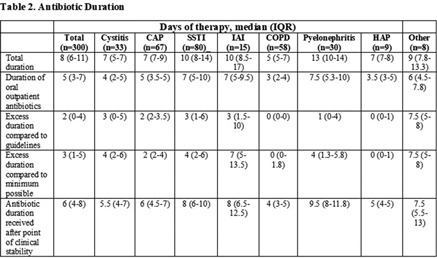

**Figure 1.**